# From across the Seas

**Published:** 1917-11-17

**Authors:** 


### FROM ACROSS THE SEAS.

### Official Journals of Public Ministries.

Our *National Food Journal* is not the only publication issued directly by a public Ministry. The Minister of Public Health of New Zealand, the Hon. G. W. Russell, commenced the monthly issue of the *Journal of the Department of Public Health, Hospitals, and Charitable Aid* in July last with the object of providing information in connection with the work of the department for the information of the members and staffs of hospital boards and other persons likely to be interested in hospital and public health matters. Mr. Russell, as the readers of this page will know, is a very live Minister, and the contents of his Journal are both interesting and valuable. The first number contains a comprehensive table of the salaries and emoluments paid by New Zealand Hospital and Charitable Aid Boards to medical, nursing, and miscellaneous officers. This table has evidently been most carefully compiled, and should save hospital boards much time and trouble when questions of remuneration come before them. Amongst other good things, the August number contains a useful article on hospital book-keeping, and another on "Charitable Relief and the Law of Settlement." There are no advertisements, but each issue contains a list of positions vacant, open to medical men and nurses, in the institutions within the jurisdiction of the Ministry of Health.

### Compulsory Enlistment of Australian Doctors.

The members of the British Medical Association in Australia have been asked to say if they desire legislation for the purpose of applying compulsory enlistment of the medical profession in Australia, for service at home and abroad. The number of members voting in favour of the proposition is 1,011, out of a total of 1,361. The Federal Committee of the Association last April decided to ask the Federal Government for legislation should three-quarters of the members be in favour of enrolment. A three-quarters majority proves to fall short of this requirement by ten, but the result of the voting is so significant that, in the opinion of the *Medical Journal of Australia*, it is obvious that the matter should not be allowed to rest. The requirements of the military authorities cannot be met under the system obtaining at present, and the civil population, especially in the public hospitals, is suffering as a result of the want of organisation.

